# A Direct Comparison of Peptide Drug Delivery Systems Based on the Use of Hybrid Calcium Phosphate/Chitosan Nanoparticles versus Unmixed Calcium Phosphate or Chitosan Nanoparticles In Vitro and In Vivo

**DOI:** 10.3390/ijms242115532

**Published:** 2023-10-24

**Authors:** Ekaterina Popova, Victoria Tikhomirova, Olga Beznos, Natalia Chesnokova, Yuri Grigoriev, Michael Taliansky, Olga Kost

**Affiliations:** 1 Shemyakin-Ovchinnikov Institute of Bioorganic Chemistry of the Russian Academy of Sciences, 117997 Moscow, Russia; popova.ekaterina1995@gmail.com (E.P.); vetikhomirova@gmail.com (V.T.); michael.taliansky@hutton.ac.uk (M.T.); 2 Chemistry Faculty, M.V. Lomonosov Moscow State University, 119991 Moscow, Russia; 3 Helmholtz National Medical Research Center of Eye Diseases, 105062 Moscow, Russia; olval2011@mail.ru (O.B.); nchesnokova2012@yandex.ru (N.C.); 4 Shubnikov Institute of Crystallography, Federal Scientific Research Center Crystallography and Photonics, Russian Academy of Sciences, 119333 Moscow, Russia; ygrigoriev@mail.ru

**Keywords:** cationic carriers, chitosan, calcium phosphate particles, angiotensin-converting enzyme inhibitor, intraocular pressure, enalaprilat

## Abstract

Nanocarriers provide a number of undeniable advantages that could improve the bioavailability of active agents for human, animal, and plant cells. In this study, we compared hybrid nanoparticles (HNPs) consisting of a calcium phosphate core coated with chitosan with unmixed calcium phosphate (CaP) and chitosan nanoparticles (CSNPs) as carriers of a model substrate, enalaprilat. This tripeptide analog is an inhibitor of angiotensin-converting enzyme and was chosen by its ability to lower intraocular pressure (IOP). In particular, we evaluated the physicochemical characteristics of the particles using dynamic light scattering (DLS) and scanning electron microscopy (SEM) and analyzed their ability to incorporate and release enalaprilat. HNPs exhibited the highest drug loading capacity and both HNPs and CSNPs demonstrated slow drug release. The comparison of the physiological effects of enalaprilat-loaded CaP particles, HNPs, and CSNPs in terms of their impact on IOP in rabbits revealed a clear advantage of hybrid nanoparticles over both inorganic and chitosan nanoparticles. These results could have important mechanistic implications for developing nano-based delivery systems for other medical, veterinary, and agricultural applications.

## 1. Introduction

Nanotechnology is widely practiced in medicine, veterinary, agriculture, and many other sectors. In medicine, nanoparticles can carry antimicrobial, anti-tumor, and antiviral agents, antioxidants, anti-inflammation, and anti-stroke agents, and also serve as a magnetic resonance imaging contrast [1,2,3,4,5,6]. The veterinary applications of nanocarriers are conceptually similar to drug delivery in humans [7]. Nanocontainers also have great agricultural potential for the delivery of nutrients and various agrochemicals including pesticides, herbicides, plant growth regulators, and foreign DNA or RNA in plants [8,9,10] through leaf cuticular or stomatal pathways using foliar spraying methods [11,12,13,14]. Apart from the general use of nanocarriers in the therapy of some disorders and diagnostics, they can also be used for the treatment of some distinctive organ diseases, e.g., eye diseases. Eye drops are the most prevalent way of therapy for eye diseases because they are convenient, demand less amount of the drug and, consequently, are much less likely to cause side effects than systemic administration. However, only a small amount of the instilled drug penetrates the inner eye structures because of cornea and sclera barriers and rapid elimination of the drug from the eye surface caused by tear turnover and nasolacrimal drainage. To overcome the ocular drug delivery barriers and improve drug bioavailability, various drug delivery systems have been developed, such as emulsions, ointments, suspensions, aqueous gels, nanomicelles, nanoparticles, liposomes, dendrimers, etc. [15]. Among these, nanosize carriers are the most attractive, as they could enhance drug permeability across the blood–aqueous barrier and cornea, prolong drug contact time with ocular tissues, provide sustained drug release for a long time, and diminish the toxicity and side effects [16,17,18,19,20,21].

Apart from the development of nanocarriers containing active components for the specific treatment of ocular disorders, this “drug-loaded nanocarrier–eye” system could be considered a model system for the comparison of different nanocarriers. In particular, encapsulation of ophthalmic drugs into the nanosize calcium phosphate (CaP) particles is promising as CaP particles are available, biocompatible, biodegradable, and nonimmunogenic [22,23,24,25,26,27], and CaP particle synthesis is simple and inexpensive [28]. Previously, using CaP particles covered with cellobiose, we enhanced the effect of a tripeptide analog, lisinopril, on the intraocular pressure (IOP) [29]. However, CaP particles themselves, as well as cellobiose-covered CaP particles, are characterized by a negative surface charge [29], which could impede drug penetration into the eye since the mucin layer on the anterior eye surface is also negatively charged [30,31]. The coating of CaP particles with mucoadhesive [32,33] and positively charged biopolymer chitosan can enhance the affinity of the particles for the epithelium of the eye and increase the bioavailability of the included drug [34,35]. Our previous research has shown that chitosan-covered CaP particles (HNPs) containing another tripeptide analog, enalaprilat, demonstrated both prolongation and enhanced efficacy of physiological action [35].

It should be noted, however, that chitosan itself is a very prospective material for many applications and can be used as the main carrier [36]. Hence, it was necessary to compare the properties of pure chitosan particles (CSNPs) with HNPs and CaP particles directly in order to determine whether the advantages of hybrid particles are principally provided by the chitosan “coat” without the need for the use of the internal inorganic core. The tripeptide analog, enalaprilat, was chosen as a substance as the effects of the inclusion of enalaprilat into the particles could be followed not only in the experiments in vitro but also in vivo by its action on IOP.

Here, we conducted a systematic and comparative study of the physical characteristics, morphology, drug capacity, and cytotoxicity of three different types of nanoparticles, CaP, HNPs, and CSNPs, as well as in vitro drug release from all types of formulations and in vivo physiological effects, which demonstrated the advantages of hybrid HNPs over individual CaP particles or CSNPs.

## 2. Results and Discussion

### 2.1. Synthesis of Different Nanoparticles and Their Characteristics

The conditions of the synthesis of CaP particles and HNPs were previously described in [35]. To synthesize CSNPs, we have chosen the method of ionotropic gelation [37]. Since the properties of chitosan vary significantly depending on the molecular weight, deacetylation degree, and various modifications [33,38,39], it was necessary to choose the conditions under which the particles with optimal characteristics could be obtained. The selected types of chitosan, low molecular weight 5 kDa chitosan and 72 kDa glycol chitosan (chitosan derivative), do not require an acidic medium for solubilization. So, the particles could be obtained in neutral or only slightly acidic conditions. Previously, it was shown that pure chitosan particles could be formed under the conditions of covering CaP particles with chitosan, but only in the absence of calcium chloride [35]. It appeared, however, that enalaprilat-containing CSNPs obtained at these conditions were unstable and, therefore, required a change in synthesis conditions, namely, pH 5.0 instead of 5.7 for the 5 kDa chitosan and pH 7.1 instead of 6.8 for the glycol chitosan, as well as much higher chitosan concentrations, in three and six times, respectively.

The effectiveness of CaP particles covered with chitosan was estimated to be 13 ± 2% and 45 ± 1% for the 5 kDa chitosan and the 72 kDa glycol chitosan, respectively. The effectiveness of CSNP formation in the chosen conditions was estimated to be about 92 ± 5% for the 5 kDa chitosan and 75 ± 7% for the 72 kDa glycol chitosan. 

Each type of empty and enalaprilat-loaded particle, CaP particles, HNPs, and CSNPs, was characterized using typical DLS characteristics: ζ-potential, size, and polydispersity index (PDI). The results are shown in Table 1.

**Figure 1:**
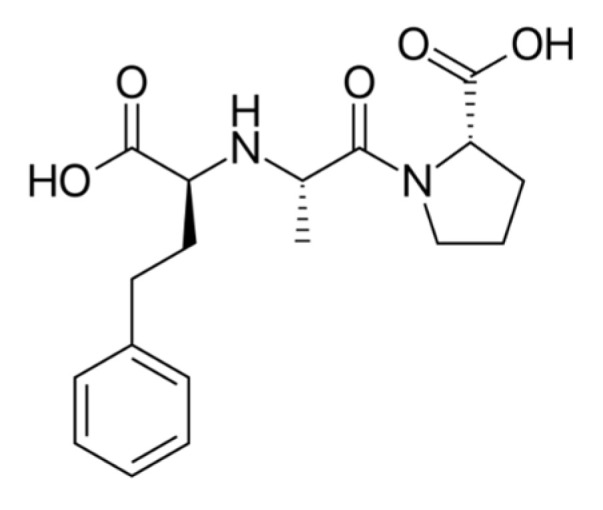
Enalaprilat structure.

While CaP particles were characterized based on negative ζ-potential, the chitosan covering resulted in a positive ζ-potential for both empty and enalaprilat-loaded HNPs. Empty CSNPs and HNPs formed with 72 kDa glycol chitosan had similar positive ζ-potential, while CSNPs formed with 5 kDa chitosan were characterized based on higher positive ζ-potential than the corresponding HNPs (Table 1). The inclusion of enalaprilat into the particles usually decreased the value of the ζ-potential, which was likely due to the presence of two carboxylic groups in the molecule structure (Figure 1). These carboxylic groups are able to coordinate with positively charged calcium ions during inorganic core formation and with positively charged amino groups in the chitosan cover.

The size of empty chitosan and hybrid particles was quite similar (Table 1). The inclusion of enalaprilat into the particles did not alter the size significantly except for chitosan particles formed with 72 kDa glycol chitosan for which enalaprilat-containing CSNPs were almost two times larger than the empty ones (Table 1). This size increase together with a drop in the ζ-potential affected the shelf life of CSNPs formed with 72 kDa glycol chitosan. While hybrid particles and CSNPs formed with 5 kDa chitosan were stored unchanged for at least a month at 4 °C, CSNPs formed with 72 kDa glycol chitosan retained their characteristics for only 2–3 weeks.

In summary, the production of chitosan particles seems to be simpler than hybrid ones, since it bypasses the stage of CaP particle core synthesis and does not require the use of a sonicator. However, the cost of CSNPs is higher than HNPs as the chitosan concentrations required for the synthesis of CSNPs are much higher than for the chitosan coating of hybrid particles. Nevertheless, all obtained particles are sufficiently stable when stored in suspension at 4 °C and have a size and ζ-potential that allow them to be considered as prospective carriers of different substances.

### 2.2. Particle Morphology

**Figure 2:**
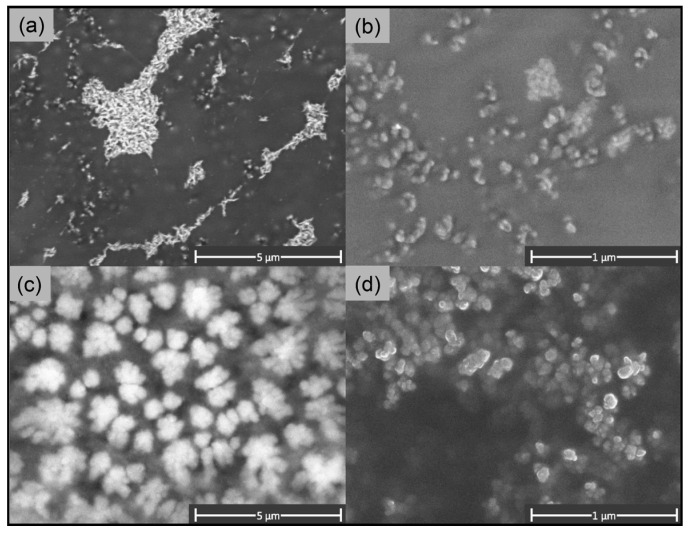
SEM images of (**a**) hybrid particles covered with 5 kDa chitosan; (**b**) chitosan particles formed with 5 kDa chitosan; (**c**) hybrid particles covered with 72 kDa glycol chitosan; and (**d**) chitosan particles formed with 72 kDa glycol chitosan.

It was shown previously that, at the chosen synthesis conditions, CaP particles have a “rod” shape [35]. Empty hybrid particles with a CaP core, formed with 5 kDa chitosan, had a rounded “rod” shape as well (Figure 2a), which coincided with the shape of uncoated CaP particles. As the coverage of hybrid particles with 5 kDa chitosan was about 13%, it did not lead to a change in the shape of the particles. However, the coverage of CaP particles with 72 kDa glycol chitosan changed the shape of the particles to a “ball” one (Figure 2c). We explain this phenomenon by the high efficacy of the covering, about 45%, and meanwhile by the high molecular weight of the glycol chitosan equal to 72 kDa. Such large molecules being on the surface of the inorganic core can obscure it.

SEM images of empty chitosan particles formed with 5 kDa chitosan demonstrated that CSNPs had an irregular spherical shape (Figure 2b). The diameter of these CSNPs determined using SEM varied from 50 to 160 nm with an average size of about 80 nm, which approximately corresponded to the mean hydrodynamic diameter determined using DLS (Table 1). The chitosan particles formed with 72 kDa glycol chitosan also demonstrated the “ball” shape (Figure 2c,d). The size of glycol chitosan particles in the images varied from 60 to 250 nm with an average value of 160 nm, which appeared to be less than the values of the average hydrodynamic diameter obtained via the DLS method (Table 1). It should be noted that all types of particles formed large agglomerates when freeze-dried on the matrix, though glycol chitosan particles were less prone to form aggregates (Figure 2d).

### 2.3. Enalaprilat Loading into the Particles

Enalaprilat was loaded onto the calcium phosphate particles in situ at the stage of CaP core formation [35]. The same approach was used for the incorporation of enalaprilat into chitosan particles, applying it directly during chitosan particle preparation. As for the hybrid particles, CaP particles were covered with chitosan in the presence of enalaprilat as we did not wash out free enalaprilat from the enalaprilat-containing CaP particles. 

While all nanoparticles, inorganic CaP particles, hybrid HNPs, and chitosan CSNPs demonstrated rather good enalaprilat capacity, enalaprilat loading into CSNPs formed with 5 kDa chitosan appeared to be the smallest, only 25 ± 4% (Table 1). The enalaprilat capacity of CSNPs formed with 72 kDa glycol chitosan was similar to that of inorganic CaP particles and higher than CSNPs formed with low molecular weight chitosan. The incorporation of enalaprilat into HNPs was 2.6 ± 0.2 times more than into CSNPs formed with 5 kDa chitosan and 1.7 ± 0.1 times more with 72 kDa glycol chitosan. As a result, both HNPs demonstrated similar enalaprilat capacity (Table 1). Apparently, the enalaprilat capacity of HNPs is higher because enalaprilat molecules can be incorporated directly into the porous structure of the phosphate core as well as into the chitosan “coat”.

### 2.4. Enalaprilat Release from Calcium Phosphate, Chitosan, and Hybrid Particles

The kinetics of an active agent release from a carrier is an important parameter for its further use since it can affect the kinetics in the target tissue/organism and, therefore, influence its efficacy. Enalaprilat release from CaP particles, HNPs, and CSNPs was studied in 150 mM of NaCl solution to simulate ionic strength typical for most biological fluids. The particles were preliminarily filtered to remove an excess of free enalaprilat from the suspension and then incubated for different time periods in a fresh portion of NaCl solution. Enalaprilat release was monitored by its ability to inhibit the control ACE.

**Figure 3:**
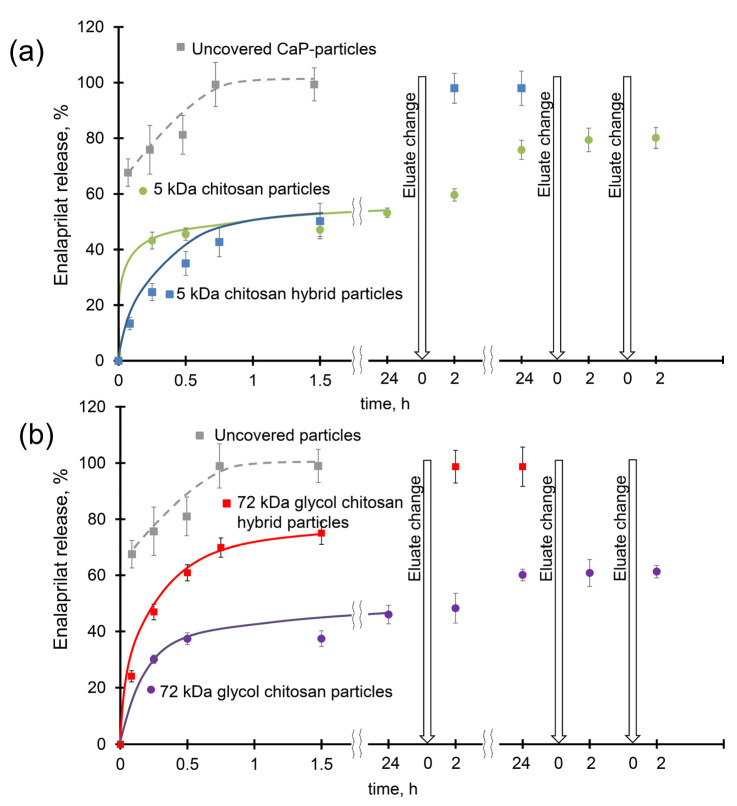
Elution of enalaprilat from CaP particles (grey hatched); (**a**) hybrid (blue) and chitosan (green) particles formed with 5 kDa chitosan; and (**b**) chitosan (grey) and hybrid (red) particles formed with 72 kDa glycol chitosan 0.15 M NaCl, pH 7.5. The results presented are the average of three analyses ± standard deviation.

The enalaprilat release profile from uncovered CaP particles showed a burst (fast release of about 60%) within the first 15 min followed by a sustained release reaching a plateau corresponding to the full release in less than an hour (Figure 3). Enalaprilat release from CSNPs formed with 5 kDa chitosan also demonstrated a burst (about 40–45%) in the first minutes followed by smooth release reaching a plateau corresponding to about half of enalaprilat incorporated into CSNPs after 1.5 h (Figure 3a). Almost full release (about 80%) was only achieved with several changes of saline. Enalaprilat release from CSNPs formed with 72 kDa glycol chitosan was also accompanied by a burst at about 30% and reached a plateau at 40% after 1 h. Subsequent washing with saline resulted in only a 65% release of enalaprilat loaded into these CSNPs (Figure 3b). Likely, enalaprilat bearing two carboxylic groups in its structure forms very tight bonds with positively charged amino groups of chitosan within CSNPs, preventing its release into the medium. Apparently, the initial burst could be explained by a release of enalaprilat located on the surface of CSNPs while the diffusion of the drug from the internal volume of the particles is hindered.

Enalaprilat release from hybrid particles was smooth without significant burst during the first minutes (Figure 3). After 90 min of incubation, almost the same amount of loaded enalaprilat was released from CSNPs (approximately 45%) and HNPs (about 50%) formed with 5 kDa chitosan (Figure 3a). However, enalaprilat release from HNPs formed with 72 kDa glycol chitosan was faster than from CSNPs (about 75% after 1.5 h) and the change in saline resulted in the full release of the drug (Figure 3). This observation could indicate that the interactions of enalaprilat with inorganic CaP core in hybrid particles were weaker than with chitosan particles.

The mathematical model that fitted the enalaprilat release from both types of CSNPs was the Korsmeier–Peppas model, as well as for hybrid particles [35]. The n-values for CSNPs formed with 5 kDa chitosan and 72 kDa glycol chitosan were equal to 0.04 and 0.08, respectively. Thus, the release of enalaprilat from CSNPs, as well as from CaP particles with *n* equal to 0.1 [35], was characterized using Fickian diffusion. The HNPs were characterized using non-Fickian diffusion and the *n*-value was equal to 0.47 for those covered with 5 kDa chitosan and 0.53 for the 72 kDa glycol chitosan. A higher *n*-value reflects a higher enalaprilat capacity. Indeed, the percentage of enalaprilat incorporation into the particles increased from the 5 kDa chitosan particles (25%) to the hybrid glycol chitosan-covered particles (72%), and the *n*-value increased from 0.04 to 0.53.

Thus, chitosan particles have a lower enalaprilat capacity, but at the same time provide a more sustained release of the loaded drug compared with hybrid particles. However, the full release of enalaprilat from chitosan particles has not been seen so far.

Since it was impossible to evaluate the effectiveness of the carriers based on the results of in vitro experiments, we analyzed their direct physiological effect on a previously used system based on the fact that the loaded enalaprilat is able to reduce IOP.

Based on the in vitro results, both CSNPs and HNPs may be selected as the most promising systems.

### 2.5. Irritation Potential

We evaluated the cytotoxicity of enalaprilat-containing CaP particles, HNPs, and CSNPs, as well as enalaprilat itself, towards erythrocytes. It is known that erythrocytes subjected to adverse external environmental changes will swell or even leak their contents. So, we could assess the potential cytotoxicity of all formulations based on their ability to cause swelling and damage to these red blood cells with the release of cellular content that produces increased levels of free hemoglobin [40]. The supernatant of a positive control group was bright red without precipitate on the bottom of the centrifugation tube, indicating the majority of erythrocytes were destroyed by extensive hemolysis, while the supernatant of a negative control group was almost clear with intact erythrocytes on the bottom. In order to calculate the degree of the putative hemolysis (HR) for free enalaprilat and CaP particles, HNP and CSNP suspensions we used as shown in Equation (1):

HR (%) = (OD_test_ − OD_neg_)/(OD_pos_ − OD_neg_) × 100%, (1)

According to the Standard Practice of the American Society for Testing and Materials Designation (ASTMF-756-17) [41], free enalaprilat is considered non-hemolytic (Table 2) as its hemolysis ratio is negligible. While for enalaprilat-loaded CaP particles, HNPs of both types and CSNPs formed from 5 kDa chitosan, the hemolysis ratios were between 2% and 5%, which meant that these formulations may be considered slightly hemolytic. However, the hemolysis ratio for CSNPs formed with glycol chitosan was above 5% (Table 2), which meant that this formulation is severely hemolytic at the present concentration. Chitosan itself can lead to erythrocyte membrane destruction [42], and since its content in the suspension of glycol chitosan particles was higher than in hybrid ones, chitosan particles could cause hemolysis. Note, however, that the main contribution to the destruction of erythrocytes by the suspension of nanoparticles was apparently caused by the low ionic strength of these suspensions. 

The instillation of the proposed formulations into the eye leads to their instantaneous dilution with lacrimal fluid, so we also tested diluted samples. The 5- and 10-time dilution of the tested samples with PBS led to the complete absence of hemolysis, even for CSNPs formed with glycol chitosan (Table 2). 

The assessment of the local irritating and allergic effect of enalaprilat and of enalaprilat-loaded CaP particles, HNPs, and CSNPs was carried out after a single instillation of preparations (short-term test), as well as after repeated instillations three times a day for 14 days (long-term test). Biomicroscopy was performed 15 min, 24 h, and 48 h after the last instillation. A score equal to 0 showed the absence of irritation; 1—weakly expressed; 2—moderately expressed; and 3—pronounced irritation. Biomicroscopy did not reveal any symptoms of irritation or allergy caused by any formulation, that is, all scores for every tested formulation were equal to 0 for the following symptoms: edema and hyperemia of the eyelids; edema and hyperemia of the conjunctiva; follicular reaction; perilimbal injection; the presence of marginal dotted infiltrates in the cornea; blepharospasm; and tearing. In summary, all the proposed nanocarriers demonstrated excellent biocompatibility in terms of cytotoxicity and irritation.

Since it was impossible to evaluate the effectiveness of the carriers based on the results of in vitro experiments, we analyzed their direct physiological effect in vivo based on the fact that enalaprilat is able to reduce IOP.

### 2.6. Effects of Topical Instillations of Enalaprilat within Calcium Phosphate, Chitosan, and Hybrid Particles on the Intraocular Pressure

We compared the effects of enalaprilat within CaP particles, CSNPs, and HNPs on IOP. Note that we did not remove an excess of enalaprilat after the preparation of any suspension of the particles. Thus, the physiological effect should be attributed to the mutual action of enalaprilat incorporated into the particles and free enalaprilat in the suspension.

In each series of experiments, the animals were randomly divided into four experimental groups receiving: (1) single instillation of the enalaprilat-loaded CSNPs; (2) single instillation of the enalaprilat-loaded HNPs; (3) single instillation of the enalaprilat-loaded CaP particles; and (4) single instillation of phosphate buffer. 

**Figure 4:**
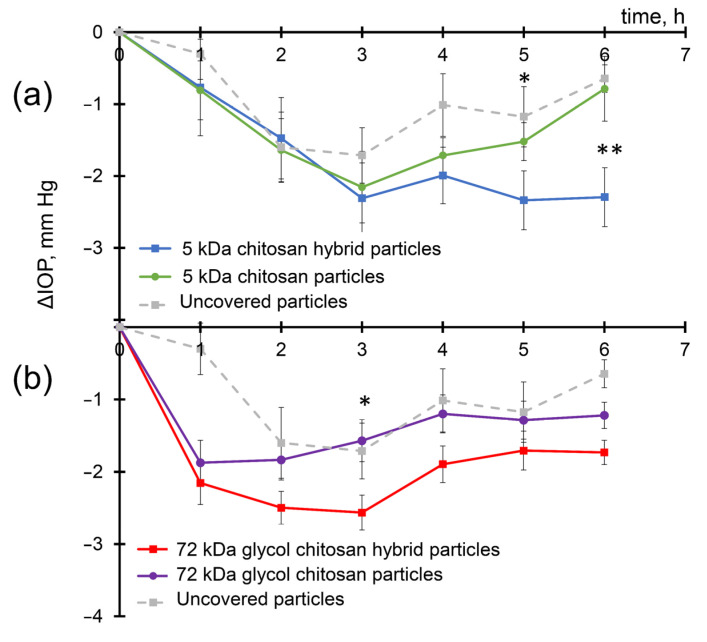
IOP decrease in normotensive rabbits after single instillations of enalaprilat within CaP particles (gray hatched); hybrid (blue and red) and chitosan (green and violet) particles formed with (**a**) 5 kDa chitosan; and (**b**) 72 kDa glycol chitosan. Enalaprilat concentration of 2.2 mM. The Mann–Whitney U test was applied to identify differences between HNPs and CSNPs. *—*p* < 0.05; **—*p* < 0.01.

Instillations of enalaprilat-loaded CaP particles led to a gradual decrease in IOP demonstrating the penetration of enalaprilat into the anterior chamber of the eye (Figure 4) in accordance with previous observations [35]. The maximum IOP decrease was about 1.7 mmHg, which occurred 3 h after instillation and lasted up to 5 h. However, the use of a chitosan coating of CaP particles or the use of chitosan particles made it possible to further increase the effect (Figure 4). For CSNPs formed with 5 kDa chitosan, the IOP minimum was also observed 3 h after instillation, but the overall effect was more pronounced (Figure 4a). As for enalaprilat-loaded HNPs formed with 5 kDa chitosan, not only was the IOP decrease more significant but the effect lasted longer (Figure 4a). 

Enalaprilat within CSNPs formed with 72 kDa glycol chitosan caused a very fast IOP decrease of up to 2 mmHg; later, the effect was comparable to that of enalaprilat within CaP particles (Figure 4b). The most remarkable effect of the inclusion of enalaprilat within particles was observed for HNPs for which the IOP decrease was found to be statistically significant at 2.6 mmHg 3 h after instillation. 

Moreover, while IOP in the experimental groups receiving enalaprilat within uncovered CaP particles and within CSNPs formed with 5 kDa chitosan returned to the initial values 6 h after the instillation (Figure 4a), the IOP in the groups receiving enalaprilat within HNPs formed with 5 kDa chitosan and enalaprilat within CSNPs and HNPs formed with 72 kDa chitosan remained significantly decreased even up to 6 h after a single instillation of the drug (Figure 4).

The difference between the efficacies of various enalaprilat-loaded particles is even better seen if the area under the curve values were considered (Figure 4, Table 3). The efficacy of the suspension of enalaprilat-loaded CaP particles was 1.9 times higher than that of free enalaprilat in solution (data for free enalaprilat not shown). The efficacy of enalaprilat-loaded CSNPs was 1.4 times and 1.2 times higher for the 5 kDa chitosan and the 72 kDa glycol chitosan, respectively, than for enalaprilat-loaded CaP particles (Table 3). The efficacy of enalaprilat-loaded HNPs covered with both the 5 kDa chitosan and the 72 kDa glycol chitosan was 1.7 times higher than enalaprilat-loaded CaP particles even when compared in a period of 6 h. 

Direct comparison of the area under the curve values obtained for enalaprilat-loaded CSNPs and HNPs demonstrated that enalaprilat within hybrid particles was at least 1.2 times and 1.4 times more effective than within chitosan particles (data are calculated for the 5 kDa chitosan and the 72 kDa glycol chitosan, respectively). Note again that these calculations were made for a period of 6 h, while the effect of reducing IOP after CSNPs and HNPS lasts longer.

This study demonstrated perspectives of hybrid nanoparticles consisting of an inorganic calcium phosphate core covered with chitosan as promising carriers of active reagents, such as ACE inhibitors. 

## 3. Materials and Methods

### 3.1. Materials

All chemicals were of analytical grade and used without further purification. Glycol chitosan (deacetylation degree ≥ 60%, molecular mass 72 kDa), low molecular weight chitosan (lactate, deacetylation degree > 90%, average molecular mass 5 (4–6) kDa), *o*-phthalaldehyde, and N-acetyl-L-cysteine were purchased from Sigma-Aldrich (St. Louis, MO, USA); sodium tripolyphosphate (STPP) was from Acros Organics (Fair lawn, NJ, USA); enalaprilat ((2S)-1-[(2S)-2-{[(1S)-1-carboxy-3-phenylpropyl]amino}propanoyl]pyrrolidine-2-carboxylic acid) was from the U.S. Pharmacopeial Convention (Rockville, MD, USA); and N-carbobenoxy-L-phenylalanyl-L-histidyl-L-leucine (Cbz-Phe-His-Leu) was purchased from Bachem AG (Bubendorf, Switzerland). Other chemicals and reagents were from Reakhim (Moscow, Russia). Angiotensin-converting enzyme (ACE) was purified from bovine lung using affinity chromatography on lisinopril–Sepharose as in [43,44]. Ultrapure deionized water was used for all experiments.

Before the synthesis, all the solutions were filtered using 0.45 μm syringe filters (Merck Millipore, Darmstadt, Germany), and the STTP solution was filtered using 0.2 μm filters (Merck Millipore).

### 3.2. Synthesis of Calcium Phosphate Particles

CaP particles were prepared as described previously [35]. Briefly, a mixture of 12.5 mM of potassium phosphate and 15.6 mM of sodium citrate solutions (5:1 *v*/*v*) was prepared. Then, a 12.5 mM calcium chloride solution (5 volume) was added simultaneously with the start of ultrasonic treatment (Bandelin Sonopuls, Berlin, Germany) for 20 min at 200 W.

### 3.3. Synthesis of Hybrid Particles

The HNPs were prepared from CaP particles with a subsequent covering of the CaP particles with chitosan as previously described [35]. The chitosan cover was produced by the addition of 1 mg/mL of chitosan solution (1 volume) to the CaP particle suspension (2 volumes) with stirring on a magnetic stirrer (400 rpm). The pH of the mixture was adjusted to 5.7 for the 5 kDa chitosan and 6.8 for the 72 kDa glycol chitosan. Next, the aqueous solution of 1 mg/mL STPP (chitosan:STTP ratio 1:0.2) was added dropwise, and the mixture was left stirring overnight. The resulting suspension of hybrid particles was stored at 4 °C.

### 3.4. Synthesis of Chitosan Particles

CSNPs were obtained via slightly modified ionotropic gelation with STTP [37]. Solutions of 1 mg/mL of 5 kDa chitosan, pH 5.0, and 2 mg/mL of 72 kDa glycol chitosan, pH 7.1, were used. A chitosan: STTP ratio of 1:0.2 was chosen for the optimal characteristics of the formed CSNPs. The 1 mg/mL STTP solution was added dropwise to the chitosan solution with constant vigorous stirring on a magnetic stirrer. The mixture was left stirring overnight. The resulting suspension of chitosan particles was stored at 4 °C.

### 3.5. Estimation of Chitosan Content

The efficiency of the formation of CSNPs and the covering of CaP particles with chitosan was evaluated as follows: An amount of 500 µL of a suspension of the particles was placed on the filters (Sartorius, Göttingen, Germany) with a pore size of 30 kDa for the 5 kDa chitosan and 100 kDa for the 72 kDa glycol chitosan and then concentrated at 5400 g to a minimum volume (25 µL). The concentrated suspension was then diluted up to 100 µL with deionized water. The concentration of chitosan in the suspension was determined by the reaction of the interaction of free amino groups of chitosan with *o*-phthalaldehyde and N-acetyl-L-cysteine with the formation of a chromophore compound [45]. The optical absorption of the solution was measured using an Infinite M200 multifunctional reader (Tecan, Männedorf, Austria) at a wavelength of 340 nm at 40 min after the start of the reaction. 

The efficiency of the formation of chitosan particles and the efficiency of the chitosan covering of the CaP particles were calculated as the ratio of the mass of chitosan in the sample to the total mass introduced during the synthesis of the particles or the covering procedure.

### 3.6. Synthesis of Enalaprilat-Loaded Calcium Phosphate, Hybrid, and Chitosan Particles

Enalaprilat (Figure 1) was loaded into CaP particles and HNPs via co-precipitation at the stage of CaP particle formation according to the protocol described above. Enalaprilat at a concentration of 7.7 mM was preliminarily dissolved in 12.5 mM of K_2_HPO_4_ solution.

The enalaprilat was also loaded into CSNPs at the stage of the CSNPs synthesis described above. The 2.64 mM solutions of enalaprilat in 1 mg/mL of 5 kDa chitosan, pH 5.0, or in 2 mg/mL of 72 kDa glycol chitosan, pH 7.1, were mixed with STTP as described above. 

Note that we did not remove the unbound enalaprilat from enalaprilat-containing particles. The final concentration of enalaprilat in CaP particle, HNP, and CSNP suspensions was 2.2 mM.

### 3.7. Dynamic Light Scattering (DLS) and Zeta Potential Measurements

All the characteristics of the obtained particles, mean hydrodynamic diameter, polydispersity index (PDI), and ζ-potential, were determined at 25 °C on a Zetasizer Nano ZS instrument (Malvern Co., Ltd., Malvern, UK). All data provided were the average of the triplicate. The hydrodynamic diameter and PDI were measured via dynamic light scattering in 6 mM of KCl solution using a backscatter angle of 173°. The size distribution was processed using non-negative least squares analysis (NNLS) (Malvern Co., Ltd.). To measure the ζ-potential, the sample was transferred into a distilled water solution. The analysis was conducted on the specialized cuvette with golden electrodes. The data were automatically processed using the Zetasizer v.7.03 software. 

### 3.8. Morphology of Hybrid and Chitosan Particles

The morphology and the shape of HNPs and CSNPs were studied using scanning electron microscopy (SEM) using a dual beam, field emission scanning electron-ion microscope Scios (1–30 kV) (FEI, Hillsboro, OR, USA). The samples were pre-dialyzed against deionized water and freeze-dried on conductive double-sided carbon tape.

### 3.9. Determination of Enalaprilat Loading

The suspensions of CaP particles, HNPs, and CSNPs containing enalaprilat were concentrated 10 times via centrifugation on a 30 kDa membrane at 5400× *g* for 5 min. The amount of unbound enalaprilat was evaluated in the filtrates based on its ability to inhibit the control ACE [35]. The catalytic activity of ACE was measured using a fluorimetric assay with 0.2 mM of Cbz-Phe-His-Leu as a substrate [46] in 0.15 M of phosphate buffer, pH 7.5, containing 0.15 M of NaCl and 1 μM of ZnCl_2_, at 25 °C, on an Infinite M-200 reader (Tecan). The enalaprilat content in filtrates was calculated using the calibration curve of the control enalaprilat as described in [35].

### 3.10. In Vitro Enalaprilat Release from Calcium Phosphate, Hybrid, and Chitosan Particles

The enalaprilat-containing CaP particle, HNP, and CSNP suspensions were divided into portions of 500 μL and concentrated 10 times on separate 30 kDa membranes at 5400 g for 5 min to remove 90% of the unbound enalaprilat. After that, 450 μL of 0.15 M NaCl solution, pH 7.5, was added to each concentrate, and the resulting suspension was incubated for various periods at room temperature. Then, the suspensions were again centrifuged and the amount of released enalaprilat was determined in each filtrate. Each experiment was performed at least three times, and the results are the mean with standard deviations.

### 3.11. Irritation Tests

All experiments with live rabbits were carried out in strict accordance with the Association for Research in Vision and Ophthalmology (ARVO) statement for the Use of Animals in Ophthalmic and Vision Research. The protocol was approved by the Local Committee on the Ethics of the Helmholtz National Medical Research Center of Eye Diseases (Permit number 22/2). In vivo studies were conducted using adult male Chinchilla rabbits weighing 2.0–2.5 kg. The animals were kept at temperatures of 21–23 °C and 50–60% humidity. Drinking water and conventional food were provided ad libitum.

#### 3.11.1. Hemolysis Assay

To assess the cytotoxicity of enalaprilat and enalaprilat-loaded CaP particle, HNP, and CSNP suspensions, we provided the hemolysis assay and evaluated the extent to which the formulations induced disruption of the membrane of erythrocytes (red blood cells). Erythrocytes were obtained from 3 mL of freshly collected heparinized rabbit blood. The blood was collected in 6 mL Sodium Heparin tubes (Guangzhou Improve Medical Instruments Co., Guangzhou, China) and centrifuged at 1500× *g* for 5 min. The supernatant was removed via aspiration and 3 mL of PBS (50 mM Na_2_HPO_4_, 150 mM NaCl), pH 7.0, was added to the remaining erythrocytes for washing. All samples were washed thrice or more until the supernatant became clear. After washing, the remaining precipitate was diluted 1:100 by resuspending 200 µL of erythrocytes in 20 mL PBS, pH 7.0, to obtain a 1% suspension of erythrocytes. For the test, 100 μL of the suspension of enalaprilat-containing nanoparticles was mixed with 100 μL of 1% erythrocyte suspension and incubated for 60 min at 37 °C in Termit thermostats (DNK-Tekhnologiya, Moscow, Russia). PBS was used as a negative control, while 10% Triton X-100 was used as a positive control as hemolysis of erythrocytes was shown to be complete in these conditions [40]. After incubation, the samples were centrifuged at 1500× *g* for 5 min, and then 100 μL of each the supernatant was transferred to a transparent, flat-bottom 96-well plate. The optical absorption was measured using an Infinite M200 multifunctional reader (Tecan) at a wavelength of 405 nm. For background absorption, PBS for experimental tests and for negative control and 10% Triton X-100 for positive controls were used. Three independent series of experiments were provided.

#### 3.11.2. Ocular Response Test

The assessment of local irritating and allergenic effects was carried out with a single and multiple administration of the test samples of enalaprilat, as well as enalaprilat-loaded CaP particle, HNP, and CSNP suspensions in comparison with the PBS, pH 7.0, as the control. The study in each series was performed on 21 rabbits. The animals were divided into 7 groups of 3 rabbits (6 eyes). Enalaprilat, CaP particle, HNP, and CSNP (using two types of chitosan) suspensions were instilled (50 µL) into both eyes of the experimental groups; PBS, pH 7.0, was instilled into the eyes of the control group.

The short-term test was performed with a single instillation. In the long-term test, the rabbits were treated with 1 dose 3 times a day within 14 days. After the last instillation in each group, all animals were examined via biomicroscopy after 15 min, 24 h, and 48 h. The biomicroscopy was carried out using an Opton 30 SL photo-slit lamp (ZEISS, Jena, Germany).

The following symptoms of the local irritant effect of the formulations were evaluated: edema and hyperemia of the eyelids, edema and hyperemia of the conjunctiva, follicular reaction, perilimbal injection, the presence of marginal dotted infiltrates in the cornea, blepharospasm, and tearing (0—the absence; 1—weakly expressed; 2—moderately expressed; and 3—pronounced).

### 3.12. Intraocular Pressure Studies

A comparative assessment of the effects of enalaprilat within different carriers on IOP was carried out on normotensive rabbits. At least three independent series of experiments for each type of enalaprilat-containing particle were performed. In each experiment, animals were randomly divided into 4 groups. The first group (5 rabbits, 10 eyes) received single instillations of 50 μL of 2.2 mM enalaprilat in CSNP suspension, pH 7.5, to each eye. The second group (5 rabbits, 10 eyes) received single instillations of 50 μL HNP suspension, pH 7.5, with the same enalaprilat concentration. The third group (5 rabbits, 10 eyes) received single instillations of 50 μL CaP particle suspension, pH 7.5, with the same enalaprilat concentration. The fourth group (3 rabbits, 6 eyes), serving as a control, received single instillations of buffer. IOP was measured before and after the instillation for 7–8 h at intervals of 1 h using a Tonovet automatic veterinary IOP monitor (Tiolat Oy, Helsinki, Finland).

### 3.13. Statistical Analysis

Data were analyzed using Statistica for Windows (version 10.0, Stat.Soft. Inc., Tulsa, OK, USA). All data are presented as means ± SD. The Mann–Whitney U test was applied with *p* ≤ 0.05 considered statistically significant and *p* ≤ 0.01 considered highly statistically significant.

## 4. Conclusions

The results of this work demonstrate that the model compound, tripeptide analog enalaprilat, can be effectively loaded into nanocarriers prepared from materials of various nature, both inorganic and biopolymer. However, nanocarriers containing chitosan, namely, pure chitosan nanoparticles (CSNPs) and hybrid nanoparticles consisting of a calcium phosphate core covered with chitosan (HNPs), have significant advantages over pure inorganic calcium phosphate nanoparticles (CaP particles) in both the capacity per loaded substance and in long-term release capability. HNPs demonstrated a higher enalaprilat capacity compared with CSNPs, but CSNPs provided a more prolonged release of the loaded drug in vitro; however, this release was incomplete.

All tested nanocarriers loaded with enalaprilat, as well as enalaprilat itself, exhibited low cytotoxicity toward erythrocytes, except for CSNPs formed with glycol chitosan, which caused quite significant damage. However, five-fold dilution with PBS canceled any negative effect of the nanoparticles. A thorough investigation of the putative irritation of the eye or inflammation after single or long-term instillation of enalaprilat-loaded nanoparticles did not reveal any negative side effects either.

Finally, the direct comparison of the physiological effects of different enalaprilat-loaded nanoparticles on rabbit intraocular pressure demonstrated clear advantages of HNPs as drug nanocarriers.

Thus, while pure CaP particles and CSNPs are prospective nanocarriers, this study showed the advantages of hybrid nanoparticles on the basis of an inorganic calcium phosphate core covered with chitosan as a nanocarrier of an active compound. Overall, the results and approaches presented here could have important mechanistic implications for developing nano-based delivery systems for various medical, veterinary, and agricultural applications.

## Figures and Tables

**Table 1:** Comparative characteristics of uncovered CaP particles, chitosan particles, and hybrid particles formed using CaP-core and chitosan.

Particle Type	Chitosan Type		ζ, mV	d, nm	PDI	% of Inclusion
CaP	-	Empty	−25 ± 1	80 ± 20	0.19	-
		Enalaprilat-loaded	−31 ± 3	110 ± 20	0.20	40 ± 10
Hybrid	5 kDa chitosan	Empty	+12 ± 2	160 ± 20	0.21	-
		Enalaprilat-loaded	+7 ± 3	180 ± 30	0.25	66 ± 5
	72 kDa glycol chitosan	Empty	+20 ± 3	240 ± 25	0.25	-
		Enalaprilat-loaded	+16 ± 4	260 ± 25	0.25	72 ± 8
Chitosan	5 kDa chitosan	Empty	+23 ± 2	110 ± 20	0.20	-
		Enalaprilat-loaded	+21 ± 3	105 ± 20	0.16	25 ± 4
	72 kDa glycol chitosan	Empty	+19 ± 2	250 ± 20	0.19	-
		Enalaprilat-loaded	+10 ± 2	460 ± 20	0.16	41 ± 3

**Table 2:** Calculated hemolysis ratios according to Equation (1) for enalaprilat and enalaprilat-loaded CaP particles, HNPs, and CSNPs on 1% rabbit erythrocyte solution. The data were averaged from three experimental replicates, each containing two technical replicates, using 10% Triton X-100 and PBS samples as positive and negative controls, respectively.

Samples	Chitosan Type	Calculated Hemolysis, %	
		Undiluted	Dilution 1/5
Enalaprilat	-	0.42 ± 0.38	0.00 ± 0.12
CaP	-	2.02 ± 0.26	0.00 ± 0.46
Hybrid	5 kDa chitosan	4.76 ± 0.47	0.00 ± 0.22
	72 kDa glycol chitosan	3.50 ± 0.44	0.00 ± 0.14
Chitosan	5 kDa chitosan	3.56 ± 0.36	0.00 ± 0.12
	72 kDa glycol chitosan	13.80 ± 1.14	0.00 ± 0.51

**Table 3:** The relative area under the IOP reduction curves under the influence of free enalaprilat and enalaprilat-containing particles. The area under the curve for free enalaprilat was taken as 100%. The concentration of enalaprilat is 2.2 mM.

**Samples**	**Chitosan Type**	**Relative Area, %**
Enalaprilat	-	100
CaP	-	190
Hybrid	5 kDa chitosan	320
	72 kDa glycol chitosan	330
Chitosan	5 kDa chitosan	260
	72 kDa glycol chitosan	240

## Data Availability

The data supporting the findings of this study are available within the article.
